# Changes in Prevalence and Seasonality of Pathogens Identified in Acute Respiratory Tract Infections in Hospitalised Individuals in Rural and Urban Settings in South Africa; 2018–2022

**DOI:** 10.3390/v16030404

**Published:** 2024-03-05

**Authors:** Michaela Davids, Siobhan Johnstone, Adriano Mendes, Gadean Brecht, Theunis Avenant, Nicolette du Plessis, Maryke de Villiers, Nicola Page, Marietjie Venter

**Affiliations:** 1 Centre for Emerging Respiratory and Arbovirus Research, Department of Medical Virology, University of Pretoria, Pretoria 0084, South Africa; 2 Centre for Enteric Diseases, Virology, National Institute for Communicable Diseases of the National Health Laboratory Service, Johannesburg 2192, South Africa; siobhanjsa@gmail.com (S.J.); nicolap@nicd.ac.za (N.P.); 3 Department of Paediatrics, Kalafong Provincial Tertiary Hospital, University of Pretoria, Pretoria 0084, South Africa; 4 Department of Internal Medicine, Kalafong Provincial Tertiary Hospital, University of Pretoria, Pretoria 0084, South Africa; 5 Centre of Enteric Diseases, Department of Medical Virology, University of Pretoria, Pretoria 0084, South Africa

**Keywords:** respiratory tract infections, multiplex real-time PCR, hospitalised cases, seasonality, COVID-19 pandemic

## Abstract

Severe acute respiratory tract infections (SARIs) has been well described in South Africa with seasonal patterns described for influenza and respiratory syncytial virus (RSV), while others occur year-round (rhinovirus and adenovirus). This prospective syndromic hospital-based surveillance study describes the prevalence and impact of public interventions on the seasonality of other respiratory pathogens during the coronavirus disease-19 (COVID-19) pandemic. This occurred from August 2018 to April 2022, with 2595 patients who met the SARS case definition and 442 controls, from three sentinel urban and rural hospital sites in South Africa. Naso/oro-pharyngeal (NP/OP) swabs were tested using the FastTrack Diagnostics^®^ Respiratory pathogens 33 (RUO) kit. Descriptive statistics, odds ratios, and univariate/multivariate analyses were used. Rhinovirus (14.80%, 228/1540) and *Streptococcus pneumoniae* (28.50%, 439/1540) were most frequently detected in NP/OP swabs and in children <1 years old (35%, 648/1876). Among others, pathogens associated with SARI cases causing disease were influenza A&B, HRV, RSV, hCoV 229e, *Haemophilus influenzae*, *Staphylococcus aureus*, and *Streptococcus pneumoniae*. Pre-COVID-19, seasonal trends of these pathogens correlated with previous years, with RSV and influenza A seasons only resuming after the national lockdown (2021). It is evident that stringent lockdown conditions have severe impacts on the prevalence of respiratory tract infections.

## 1. Introduction

Pathogens associated with acute lower respiratory tract infections have a major impact on global health [1]. These lower respiratory tract infections are one of the leading causes of global mortality and morbidity in extreme age groups, such as children younger than 5 years and the elderly aged 60 and older, leading to 2.5 million deaths in 2019 [2]. The Global Burden of Disease estimated that 89% of the 336 million pneumonia cases in 2016 were from low- and middle-income countries, and 62% of the estimated 2 million pneumonia deaths occurred in sub-Saharan Africa and South Asia [1]. Viral respiratory pathogens such as human rhinovirus (HRV), coronavirus, respiratory syncytial virus (RSV), and influenza are the major pathogens found to cause infections in the upper respiratory tract, although many others may be present [3,4]. Common bacterial respiratory pathogens may colonise in the upper respiratory tract, and the detection of these bacterial pathogens may not be associated with clinical signs [5]. Invasive pneumococcal pneumonia has been shown to be associated with high nasopharyngeal pneumococcus density and viral co-infection [6]

The major causes of influenza-like illness (ILI) and severe acute respiratory illness (SARI) in children and the elderly were shown to be respiratory syncytial virus (RSV) and influenza during sentinel surveillance studies in South Africa from 2013 to 2015 [7]. The seasonal influenza and RSV mortality rates were 23.0 (95% CI 11.0–30.6) and 13.2 (95% CI 6.4–33.8) per 100,000 people annually of all deaths, with the peak ages being ≥75 years for influenza and infants for RSV. Among children under the age of 2 years, RSV deaths were likely to occur in hospital, whereas deaths caused by influenza were likely to occur out of hospital [8]. Factors associated with an increased risk of ILI or SARI in SARI hospitalised patients and ILI outpatients include age (being <5 years old and ≥65 years) and underlying conditions such as human immunodeficiency virus (HIV) infection, pregnancy, working in mines [9]. Severe acute respiratory syndrome coronavirus 2 (SARS-CoV-2) causes SARI, mainly in adults and the elderly, and results in severe cases and mortality in immunocompromised individuals and the elderly [10]. The first case of SARS-CoV-2 in South Africa occurred in March 2020; shortly thereafter, the South African government initiated the first national lockdown period with stringent conditions, which only eased in June 2020 [11].

The African Network for Improved Diagnostic, Epidemiology and Management of Common Infectious Agents (ANDEMIA) is a transnational sentinel surveillance study [12]. This study is based on the aetiology and clinical characteristics of three syndromes—acute respiratory infections (ARIs), gastrointestinal infections (GIs), and acute febrile diseases of unknown cause (AFDUCs)—which are amongst the top five causes of death in sub-Saharan Africa [13]. The target syndrome described in the current study is ARIs.

The understanding of how the pandemic influenced the prevalence and seasonality of respiratory pathogens in hospitalised patients with ARI, as well as their contribution to disease before and during the pandemic, when compared to control patients without respiratory signs, may aid in the contribution of these pathogens’ role in respiratory diseases in South Africa. The knowledge of these respiratory pathogens’ circulation may also contribute to the possible role of public health interventions during the pandemic. Therefore, the objectives of this study were to define the contribution of respiratory pathogens to severe acute lower respiratory tract infections (SARIs) with either a current or history of fever in two geographically distinct (rural and semi-rural) areas in South Africa (Gauteng and Mpumalanga Provinces) between 2018 and 2022. Since patients were screened for SARS-CoV-2 before they entered hospitals and subsequently sent to coronavirus disease-19 (COVID-19) wards, this study did not address SARS-CoV-2 infections but rather other respiratory pathogens before and during the pandemic in hospitals in South Africa. We tested naso/oro-pharyngeal (NP/OP) swabs of hospitalised individuals for the most common viral pathogens, excluding SARS-CoV-2, as well as common and atypical bacterial pathogens to determine their contribution to acute infection or colonization. A control group was included to compare the prevalence of these pathogens in hospitalised individuals with severe acute respiratory tract infections to individuals at the same hospitals for reasons other than respiratory-related illnesses, GIs or AFDUCs. Through this study, we could describe changes in the detection rate and seasonality of respiratory viruses and bacterial pathogens prior to the COVID-19 pandemic, through the lockdown period in 2020 and after the lockdown period was lifted in 2021.

## 2. Materials and Methods

### 2.1. Study Design and Population

This study was part of the ANDEMIA study [12]. ANDEMIA was a prospective syndromic hospital-based sentinel surveillance study investigating the contributions of respiratory pathogens to hospitalizations associated with acute respiratory infections from August 2018 to April 2022. The study population included patients of all ages who were admitted to three sentinel hospitals—one in Gauteng (Kalafong) and two in Mpumalanga Province (Matiwane and Mapulaneng)—that met the severe acute respiratory infection (SARI) case definitions and provided informed consent. Patients who were admitted with an onset of symptoms within 10 days had to meet the SARI case definition. Patients were enrolled either as SARI cases (including suspected COVID-19 disease) or as both SARI and GI cases. The SARI exclusion criteria included newborn babies who were been discharged following delivery and patients transferred directly to intensive care units without passing through the admission ward. A control group was enrolled, consisting of patients from the same community attending the hospitals, outpatient department, or clinics (including vaccine, dental, and eye clinics) for conditions other than respiratory-related illnesses. These patients were enrolled across all three hospital sites from 2019 to 2022 and were selected based on showing no signs and symptoms of respiratory illness (including cough, expectoration, pulmonary consolidation, chest pain, pneumonia, or apnoea; GIs; or AFDUCs with or without neurological signs) within the previous three weeks as described in the ANDEMIA protocol [12]. Control enrolment was affected by restricted ward and clinic access to study staff and lower numbers of elective surgeries and outpatient visits during the pandemic. It was therefore not possible to frequency match (by age, site, and season) controls to cases as per the original protocol. 

### 2.2. Sample Size

A sample size of 350 patients (cases) per year, per site (Gauteng and Mpumalanga) was targeted by surveillance officers (SOs). These patients were selected from all age groups, throughout the week (Monday to Friday), until the weekly total was met, which was 7–8 patients per week. A total of 700 patients presenting to primary health facilities with illness other than respiratory signs, diarrhoea, or fever (controls) were also enrolled, which were divided between urban and rural settings. 

### 2.3. Specimen Collection and Processing

Naso/oro-pharyngeal (NP/OP) swabs were collected in the same viral transport medium from the sentinel hospitals and transported to the Centre for Emerging arbo- and respiratory virus research, Department of Medical Virology, University of Pretoria. Once these specimens were received, they were stored at 4 °C and extracted within 48 h after collection. Each specimen was submitted with a laboratory submission and case investigation form which contained demographic and epidemiological information. The specimens were aliquoted into labelled 1.5 mL Eppendorf tubes and stored at −80 °C. 

### 2.4. Nucleic Acid Extraction

For nucleic acid extraction, a spin column-based procedure using the QIAamp viral RNA mini kit (Qiagen, Hilden, Germany) was used for manual extraction according to the manufacturer’s guidelines. For automated extractions, the QIAcube (Qiagen, Hilden, Germany) was used. The nucleic acids were extracted from 200 µL of the NP/OP swabs and the extracted nucleic acids were eluted in 100 µL of elution buffer, aliquoted, and stored at −80 °C. During extraction preparation, 4 µL (per specimen) of internal control was added to the lysis buffer solution. 

### 2.5. Multiplex Real-Time PCR for Nucleic Acids Extracted from Nasopharyngeal and Oropharyngeal Swabs

The FastTrack Diagnostics^®^ (FTD) Respiratory pathogens 33 (RUO) kit (Siemens Health engineers, Erlangen, Germany) was used to detect the presence of 19 viral, 13 bacterial, and 1 fungal pathogen. This kit consists of eight multiplex reactions for the qualitative detection of 33 pathogens: influenza A virus; influenza B virus; influenza C virus; influenza A (H1N1) virus—swine lineage; human parainfluenza 1, 2, 3, and 4 (HPIV 1–4); human coronavirus NL63, 229E, OC43, and HKU1; human metapneumoviruses A and B (HMPV A&B); human rhinovirus (HRV); respiratory syncytial virus A and B (RSV A&B); human adenovirus (HAdV); enterovirus (EV); human parechovirus (HPeV); human bocavirus (HBoV); *Pneumocystis jirovecii*; *Mycoplasma pneumoniae*; *Chlamydophila pneumoniae*; *Streptococcus pneumoniae*; *Haemophilus influenzae b*; *Staphylococcus aureus*; *Moraxella catarrhalis*; *Bordetella* spp.; *Klebsiella pneumoniae*; *Legionella pneumophila/Legionella longbeachae*; *Salmonella*; and *Haemophilus influenzae*. The kit is a one-step real-time PCR whereby a 25 µL mastermix reaction is made up, comprising 25× RT-PCR enzyme mix, 2× RT-PCR buffer, and 8 different primer and probe mixes with 10 µL of nucleic acid. The following thermocycler conditions were used on the ViiA™ 7 Real-time PCR machine (Applied Biosystems, MA, USA): 50 °C for 15 min hold, 94 °C for 1 min hold, 40 cycles of 94 °C for 8 s, and 60 °C for 1 min. Thereafter, the results were analysed using the QuantStudio™ Real-time PCR Software, version 1.3 (Applied Biosystems).

### 2.6. Data Analysis and Management

The demographic and epidemiological data, as well as laboratory findings from this study, were uploaded to a Voozanoo study data base. The data were analysed with STATA 15 and Excel to determine the association between respiratory pathogens detected in NP/OP swabs collected from hospitalised patients at the Gauteng and Mpumalanga hospital sites as well as controls with demographic and clinical data collected from each patient. Descriptive statistics, odds ratios, and univariate/multivariate logistic regression analyses, controlling for site and age throughout the years, were used to describe the epidemiology of the individual pathogens. As previously described, the control enrolment was affected by the COVID-19 pandemic, and hence, controls were not frequency matched to cases. For this reason, the logistic regression analysis comparing cases and controls was unmatched, but age and site were included in the multivariate analysis.

## 3. Results

### 3.1. Enrolment and Baseline Characteristics

From August 2018 through to April 2022, a total of 3037 (2595 patients and 442 controls) were enrolled and tested (Table 1a). Some samples were not tested due to several reasons, such as insufficient sample volume, no sample collected, or missing samples, and these were excluded from this study. The distribution and demographics of cases and controls over the study years are indicated in Table 1b. The age categories covered all ages from children to the elderly, with the highest number of specimens tested in cases from patients less than 1 year old (39% (603/1540)), and in controls, patients aged between 15 and 54 years old (45% (150/336)). There was an even distribution of specimens being tested between males (48%, 736/1540) and females (52%, 789/1540). 

### 3.2. Detection of Respiratory Pathogens in NP/OP Swabs from RTI Cases

Of the RTI cases, the viral pathogen that was detected the most across the five years in NP/OP swabs was HRV (15%, 228/1540) and the bacterial pathogen detected the most in NP/OP swabs was *Streptococcus pneumoniae* (29%, 439/1540) (Table 2). There was an association between the detection of respiratory pathogens and the year of detection (*p* ≤ 0.05). RSV (19.07%, 115/603) and *Streptococcus pneumoniae* (37.3%, 225/603) were detected most frequently in cases amongst children <1 year old, followed by HRV (19.89%, 73/367) and *Haemophilus influenzae* (35.70%, 131/367) in children aged between 1 and 4 years old. In adolescents aged 5–14 years, HRV (23.88%, 16/67) and *Staphylococcus aureus* (32.84%, 22/67) were the pathogens detected the most in respiratory specimens. In patients aged 15–54 years, there were fewer viral pathogens detected than bacterial pathogens, with *Staphylococcus aureus* (4.80%, 75/1540) being the pathogen detected the most. Similarly, fewer viral pathogens were detected in NP/OP swabs of adults aged ≥55 years compared to bacterial pathogens, with *Staphylococcus aureus* (2.92%, 45/1540) being the highest. The highest number of respiratory pathogens was detected in Gauteng Province (56.18%, 2052/3652) compared to Mpumalanga (43.84%, 1601/3652). Viral respiratory pathogens associated with cases in children were HAdV, HboV, HPeV, HRV, RSV, hCoV 229e, HMPV A&B, and HPIV 3, and the bacterial respiratory pathogens were *Bordetella* spp., *Chlamydophila pneumoniae*, *Mycoplasma pneumoniae*, and *Staphylococcus aureus.* Pathogens associated with both children and adults were mainly bacterial: *Haemophilus influenzae*, *Klebsiella pneumoniae*, *Moraxella catarrhalis,* and *Streptococcus pneumoniae*.

Table 2 represents the number (n/N, %) of viral and bacterial pathogens detected in patients/cases per year (2018–2022) from NP/OP swabs.

### 3.3. Association between Age, Province, and the Prevalence of Respiratory Pathogens among Cases Relative to Controls

Cases from Gauteng Province had a higher positivity rate for respiratory pathogens (79.07%, 2052/2595) compared to cases from Mpumalanga Province (40.87%, 1061/2595). To compare the frequency of the detection of viral and bacterial pathogens in patients with SARI vs. patients attending hospitals for other procedures that did not have respiratory/GI or AFDUC infections, cases were compared with controls (Table 3). The univariate analysis and multivariant analysis were controlled for age and site. Using multivariant analysis, for the viral pathogens, amongst the cases compared to controls, only HRV, RSV, hCoV 229E, hPMV, and IBV had an increased OR with statistical significance in patients with respiratory signs. However, there was an increased OR in patients with respiratory infections being infected with the following viral pathogens compared to controls: HRV, RSV, hCoV 229E, hCoV HKU1, IAV H1N1, IFB, HMPV A&B, and HPIV-1. IAV and PIV-2 could not be calculated since no positives were detected in the controls, although they were detected in patients. HAdV and HBoV were higher in cases than controls but were only statistically significant in the univariate analysis. Bacterial pathogens that had a statistically significantly greater OR in the multivariate analysis in patients vs. controls included *Bordetella*, *Chlamydophila pneumoniae*, *Haemophilus influenzae*, *Staphylococcus aureus*, *Streptococcus pneumoniae*, and *Mycoplasma pneumoniae,* as well as the fungal pathogen Pneumocystis jirovecii (*p* ≤ 0.05). Atypical bacteria that were detected in cases but not in controls included eight cases of Legionella and six cases of Salmonella. 

### 3.4. Seasonality of Respiratory Pathogens Detected in NP/OP Swabs of Cases

**Figure 1:**
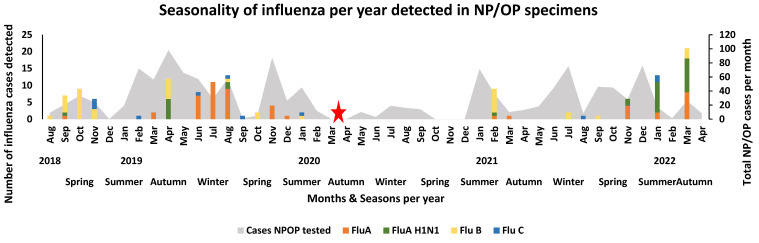
Seasonality of influenza per year detected in NP/OP specimens of hospitalised patients in Gauteng and Mpumalanga, South Africa (2018–2022). The stacked graph illustrates the number of selected viral pathogens detected per month per year. The bar graphs are colour-coded and represent the selected viruses. The area graph represents the total number of NP/OP cases that were tested per month. The red star represents the month when the first SARS-CoV-2 case occurred in South Africa, leading to the start of the pandemic in South Africa.

Influenza A cases were mainly detected during the winter season (2.07%, 27/1301) of 2019 and during the autumn season (0.6%, 8/1301) of 2022 only (Figure 1). Influenza A H1N1 cases were mostly detected during the autumn seasons of 2018 (0.46%, 6/1301) and 2019 (0.77%, 10/1301). The highest detection amongst the different influenza strains was influenza B, which was mostly detected during the spring season of 2018 (1.31%, 17/1301), the autumn season of 2019 (0.46%, 6/1301), and the summer season of 2021 (0.54%, 7/1301). Amongst the different influenza strains, influenza C was the least detected, mainly occurring in the spring season (0.23%, 2/1301) of 2018 and the winter season (0.15%, 2/1301) and summer season (0.15%, 2/1301) of 2019 and 2022, respectively (Figure 1).

**Figure 2:**
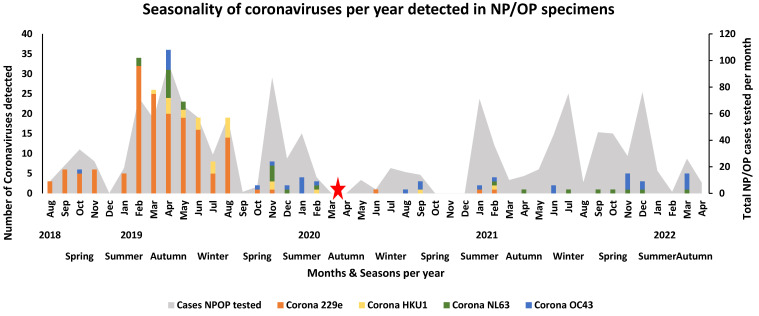
Seasonality of coronaviruses other than SARS-CoV-2 per year detected in NP/OP specimens of hospitalised patients in Gauteng and Mpumalanga, South Africa (2018–2022). The stacked graph illustrates the number of selected viral pathogens detected per month per year. The bar graphs are colour-coded and represent the selected viruses. The area graph represents the total number of NPOP cases that were tested per month. The red star represents the month when the first SARS-CoV-2 case occurred in South Africa and when lockdown was implemented and signals the start of the pandemic in South Africa.

Before the first case of COVID-19 in March 2020 in South Africa, human coronaviruses other than SARS-CoV-2, such as hCoV 229e, hCoV HKU1, hCoV NL63, and hCoV OC43, were detected during the autumn to spring seasons of 2019 (Figure 2). Before the stringent lockdown due to the SARS-CoV-2 pandemic, human coronavirus 229E was detected during the spring (1.31%, 17/1301) months of 2018 and the summer (2.84%, 37/1301), autumn (4.92%, 64/1301), and winter (2.69%, 35/1301) months of 2019. After the lockdown, lower levels of detection occurred during the summer months of 2021 (0.15% 2/1301). The human coronavirus that was rarely detected throughout the study was human coronavirus OC43, occurring mainly during the autumn (0.38%, 5/1301) season of 2019 and the summer (0.46%, 6/1301) seasons of 2019 and 2022, respectively. Human coronavirus cases were mainly detected during the autumn season (0.54%, 7/1301) and winter season (0.85%, 11/1301) of 2019. Human coronavirus NL63 was mainly detected during the autumn season (0.69%, 9/1301) of 2019. Both human coronaviruses NL63 and OC43 were detected at low levels after the pandemic (Figure 2).

**Figure 3:**
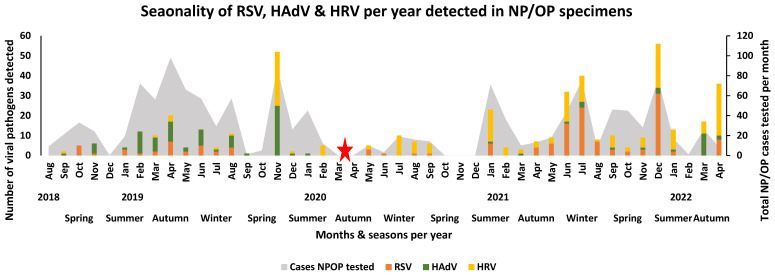
Seasonality of RSV, HAdV, and HRV per year detected in NP/OP specimens of hospitalised patients in Gauteng and Mpumalanga, South Africa (2018–2022). The stacked graph illustrates the number of selected viral pathogens detected per month per year. The bar graphs are colour-coded and represent the selected viruses. The area graph represents the total number of NP/OP cases that were tested per month. The red star represents the month when the first SARS-CoV-2 case occurred in South Africa, when lockdown occurred, and signals the start of the pandemic in South Africa.

**Figure 4:**
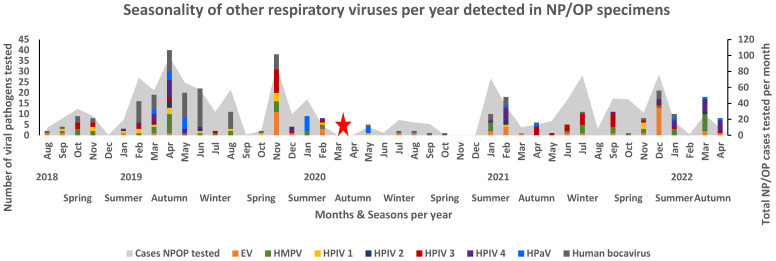
Seasonality of other respiratory pathogens per year detected in NP/OP specimens of hospitalised patients in Gauteng and Mpumalanga, South Africa (2018–2022). The stacked graph illustrates the number of selected viral pathogens detected per month per year. The bar graphs are colour-coded and represent the selected viruses. The area graph represents the total number of NP/OP cases that were tested per month. The red star represents the month when the first SARS-CoV-2 case occurred in South Africa, when lockdown occurred, and signals the start of the pandemic in South Africa.

Before the lockdown period, RSV was prevalent throughout the autumn and winter seasons of 2019, while the RSV season appeared to have started in February–March 2020 but halted when the country went into lockdown. The detection of RSV was observed the most after the lockdown periods were lifted, occurring in the winter months of June 2021 (36.3%, 16/44) and July 2021 (32.0%, 24/75) and the summer month of December 2021 (40.8%, 31/76) (Figure 3). Human rhino virus was detected in cases throughout the study period (2018–2022) (Figure 3). High peaks of HRV detection occurred during the spring season (2.08%, 27/1301) of 2019, the winter seasons of 2020 (1.23%, 16/1301) and 2021 (2.23%, 29/1301), the summer season (1.69%, 22/1301) of 2021, and the autumn season (2.46%, 32/1301) of 2022. Human adenovirus was mostly detected in cases before the SARS-CoV-2 pandemic, occurring mainly during the summer (0.85%, 11/1301), autumn (1.46%, 19/1301), and spring (2.00%, 26/1301) season of 2019. Subsequently, an increasing peak occurred during the autumn (1.00%, 13/1301) season of 2022 after the lockdown period (Figure 3). The remaining respiratory viruses (hPIV 1, hPIV 2, and hPIV 3; hMPV; enterovirus; hPaV; and human bocavirus) showed no clear seasonal distribution across the years. These viruses were mainly detected during the summer to winter months of each year, with a decline in detection during the lockdown period (Figure 4).

## 4. Discussion

This study was part of the ANDEMIA study that aimed at investigating the causes of severe acute respiratory infections in hospitalised patients with acute fever or a history of fever and acute respiratory signs that involved the lower airways, according to the WHO case definition for SARI, in South Africa, as well as countries in West Africa. This report focuses on the trends in South Africa and allowed us to describe changes in seasonality and incidence before, during, and after the COVID-19 lockdown period. Three sentinel sites were selected in South Africa: one hospital in the urban temperate region of Gauteng Province and two hospitals in the rural, drier, subtropical region of Mpumalanga Province. The advantage of this study was that it started 18 months before the onset of the COVID-19 pandemic and ended in December 2021, which allowed us to measure changes in the prevalence of other respiratory pathogens throughout the pandemic and lockdown period. Prior to the lockdown period, which started in March 2020, there was a high prevalence of viral and bacterial pathogens detected throughout 2018 and 2019 with distinct seasonal patterns. 

South Africa declared a National State of Disaster on 15 March 2020 due to the COVID-19 pandemic [14,15]. Increasing transmission of SARS-CoV-2 then led to a nationwide lockdown from 27 March 2020 to ease the burden on the health system [16]. South Africa eventually called off the National State of Disaster by 5 April 2022 [17]. This lockdown period started off with stringent conditions, whereby everyone apart from essential workers was forced to stay at home with no social activities allowed (alert level 5) and then later eased into less tight conditions, where people were allowed to return to work but with social distancing, masks, and hand sanitising remaining in place (March 2020 to September 2020) (alert level 1-4) [18]. During this period, the prevalence of viral and bacterial pathogen detection decreased extensively to almost no detection, as seen in the seasonal graphs. The prevalence of viruses such as the influenza viruses, hCoV 229E, hCoV NL63, HAdV, and RSV increased again after the stringent lockdown period was over. The multiplex FTD respiratory pathogen 33 (RUO) kit successfully detected viral and bacterial pathogens associated with respiratory infections in cases and controls from three sentinel hospitals from August 2018 to April 2022 in South Africa. This kit was used in similar studies in Africa and Asia, identifying bacterial and viral respiratory pathogens from NP/OP swabs [19,20]. This kit allowed us to investigate changes in the prevalence and seasonality of pathogens from before and during the COVID-19 pandemic in South Africa. Since it is well accepted that certain bacterial pathogens may be present in the respiratory tract through carriage rather than the cause of disease in patients, the original protocol aimed to include a subset of controls that were frequency matched based on age, site, and season to the cases. The control group included patients that had surgical procedures or attended vaccine or other clinics at the hospitals or clinics. Control enrolment was severely limited during the pandemic period, as study staff had limited access to wards and clinics, services and procedures were restricted, and fewer patients visited the hospitals and clinics. Due to these limitations, this study could not be used to define the attributable fraction of detected pathogens to disease. Another limitations of the control group was that it did not match the exact period of the study and enrolment because of the COVID-19 interruptions. The enrolment of controls started in 2019 and few cases could be enrolled during the lockdown period in 2020. Most of the controls were therefore from 2021 and cannot be used to compare seasonality of the pathogens in controls. The multivariate analysis was controlled for age and study site, only not for the period, and can only be used to identify differences in pathogens during the study period in the patient group relative to the control group, with some viruses such as influenza A not being detected at all in controls, and should therefore be interpreted with caution. The data did however correlate with previous studies carried out in South Africa, but adds data on a wider range of pathogens and the effect of regulations during the pandemic period [21,22].

Based on the detection rate, Gauteng Province, being an urban region with temperate climate conditions, detected a higher amount of pathogens compared to Mpumalanga, which is a rural region with dry, subtropical climate conditions. This correlates to a study carried out in Sweden, which shows a high detection rate of respiratory pathogens in temperate conditions [23]. Gauteng Province, being an urban region, is more populated compared to Mpumalanga, which could result in more people having access to health care facilities when needed. The majority of respiratory pathogens were detected in children, and although these pathogens do affect adults, not many were associated with adults only. 

Respiratory viruses such as RSV and influenza have a well-documented seasonal trend. The RSV season in the Southern Hemisphere starts before the influenza season, which could contribute to the severity of influenza infection [8,24]. In South Africa, amongst other Southern African countries, the influenza season occurs during the winter months of May to August [25,26]. The seasonality trends from August 2018 to March 2020 in this study were similar to previous studies. During the pandemic, a decline in the detection rate of respiratory pathogens other than SARS-CoV-2, such as influenza and RSV, was observed that correlated with the periods of lockdown restrictions [27,28]. Once the lockdown restrictions were less stringent, schools reopened, and adults returned to their workplaces, an increase in the detection rate occurred for these pathogens. This trend was also seen in other parts of the world such as France, Finland, Alaska, and Korea [26,29,30]. This lockdown period was at the onset of the RSV and influenza A seasons, and as a result, the RSV season was delayed until May 2020, while the influenza season was delayed for the next 2 years. The RSV season was likely delayed by the lockdown, since schools and daycare centres were closed and children were kept at home during lockdown. Once the lockdown was lifted, a delayed RSV outbreak occurred in May 2020. Other countries in Africa, such as Burkina Faso, reported the detection of influenza in different months compared to South Africa, affecting mostly children and correlating to the Northern Hemisphere influenza season [31]. Influenza A is seeded every year from the Northern Hemisphere in South Africa and usually starts in May and carries on through the winter season [32]. With the lockdown in 2020, before the influenza season and continued travel restrictions, it likely further delayed introduction into South Africa. The detection of influenza B started in August 2018 which occurred after the influenza season but was then detected during the second COVID-19 wave (November 2020 to February 2021). Human coronaviruses other than SARS-CoV-2, such as hCoV 229E, hCoV HKU1, hCoV NL63, and hCoV OC43, are known to cause mild cold infections yearly, with regular re-infections. These coronaviruses are also associated with SARIs and had a high detection rate prior to March 2020 in this study, which was during the SARS-CoV-2 lockdown in South Africa [33]. HCoV OC43 was detected at a very low rate during the lockdown period and continued to be detected after the lockdown period at a lower rate. The control measures during the pandemic, such as lockdown, the washing of hands with 75% ethanol, and mask wearing, may have contributed to containing the spread of these other coronaviruses. Respiratory viruses such as HRV and HAdV and bacterial pathogens that are known to be present throughout the year did not change as drastically in this study. The year-round detection of HRV and HAdV correlated with a study carried out by our group, 10 years before this study, in the Tshwane region, which showed the contribution of respiratory pathogens to annual hospitalization in South Africa [34]. The lack of impact of the interventions during the pandemic on HRV and HAdV may be because these viruses are less sensitive to the ethanol disinfection used during the COVID-19 pandemic due to a lack of a lipid envelope [10]. HRV was also shown to occur year-round in Brazil, with an increase during the winter seasons and mainly affecting children [35]. Previous studies also suggested no indication of seasonality of HAdV in South Africa and Japan [36,37]. In Japan, EVs were more frequently detected during the summer months while hMPVs had defined seasons in autumn and winter, whereas, in this study, there was a high prevalence during the autumn and spring months. Bacterial pathogens such as *Streptococcus pneumonia* and *Haemophilus influenza* are known to cause invasive diseases worldwide, with high incidence rates detected by the IRIS network prior to the COVID-19 pandemic and then decreases during the COVID-19 pandemic period [38]. This corresponds to our study, which showed a similar decline in the detection of these two pathogens during the lockdown period.

Despite the limitations of our study, atypical bacterial pathogens as well as the viruses described here, which showed a positive correlation with cases, correlated with findings from a large multi-country study which analysed NP-OP specimens from children with severe pneumonia in hospital admission relative to controls, where similar viruses such as RSV, HRV, and HMPV A&B showed a high association with disease [20]. The increased detection of bacterial pathogens known to be colonisers, such as *Haemophilus influenzae*, *Klebsiella pneumoniae*, *Streptococcus pneumoniae*, and *Staphylococcus aureus*, in patients with respiratory disease should, however, be interpreted with caution.

The limitations of this study were mainly centred on the SARS-CoV-2 pandemic. During the COVID-19 pandemic, patients were screened for SARS-CoV-2 before they were admitted to hospital, and this therefore excludes patients with SARS-CoV-2 co-infection, which could result in a decline in patients enrolled and tested during the lockdown period. The lower detection of cases could also be related to patients avoiding hospitals during this time despite being sick. Subsequently, the decline in cases may have impacted the true representation of respiratory pathogens circulating within the area and also created uncertainty as to whether these patients were severely ill or not during the lockdown period. Another limitation of this study was not enrolling enough age-matched controls in order to conduct a thorough case–control surveillance study, and the enrolment of controls may not have been from the same period as that of the cases, which prevented further analysis on seasonality between the controls and cases. Another limitation of this study was that although the multiplex assay detected common bacterial pathogens in the respiratory tract, some are known colonisers, and without blood cultures, they cannot be conclusively attributed as the cause of the disease. 

## 5. Conclusions

The detection of respiratory pathogens using multiplex real-time PCR assays is advantageous in defining multiple aetiologies of disease. These assays play a crucial role in a setting as they provide insight into what pathogens are circulating and how each pathogen influences the next in terms of seasonality and detection. In South Africa, not many studies have shown the effect that the lockdown period had towards the prevalence of other respiratory pathogens. Testing for other respiratory pathogens decreased in routine diagnostic settings due to the focus on COVID-19 testing. This study provides insight on changes in the seasonality and circulation of well-described pathogens during the pandemic. It is evident that the lockdown period with stringent conditions, such as wearing masks, social distancing, and remote schooling and working from home, as well as travel restrictions, slowed down the transmission of viruses and bacteria circulating within the two communities. The overall detection of the viral and bacterial pathogens correlated to similar studies in South Africa. The regulations implemented throughout the COVID-19 pandemic impacted the seasonality and prevalence of some of the most common viruses, such as RSV and influenza, and changed the landscape of pathogens from 2020 to 2022 in South Africa.

## Figures and Tables

**Table 1:** Overall number of cases and controls enrolled and tested and demographic characteristics of cases tested in Gauteng and Mpumalanga, South Africa, 2018–2022.

1a. Overall patients enrolled and tested				
		Cases (%)	Controls (%)	Total
**Patients enrolled**		3121	939	4060
**Patients tested**		2595	442	3037
**NP/OP**	Patients enrolled	1713 (55)	482 (51)	2195
	Specimens tested	1540 (52)	336 (76)	1876
**1b. Demographic characteristics of individuals with NP/OP specimens tested for respiratory pathogens**				
		Cases, N = 1540 (%) ^a^	Control, N = 336 (%) ^a^	Total
**Province**	Gauteng	789 (51)	174 (52)	963
	Mpumalanga	751 (49)	162 (48)	913
**Year**	2018	178 (11.5)	0 (0)	178
	2019	634 (41)	46 (14)	680
	2020	308 (20)	42 (12)	350
	2021	412 (27)	174 (52)	586
	2022	8 (0.5)	74 (22)	82
**Age**	<1 years	603 (39)	45 (13)	648
	1–4 years	367 (24)	60 (18)	427
	5–14 years	67 (4)	57 (17)	124
	15–54 years	277 (18)	150 (45)	427
	≥55 years	222 (14)	24 (7)	246
**Gender**	Male	736 (48)	176 (52)	912
	Female	789 (51)	158 (47)	944

1a—Overall patients that were enrolled and tested with their corresponding specimen type, NP/OP for both cases and controls. 1b—Baseline characteristics of the patient NP/OP specimens that were tested amongst the controls and case. ^a^ Data represents no. (%) of patients/cases and controls.

**Table 2:** Percentage of respiratory pathogens detected in NP/OP specimens tested per year from hospitalised patients in Gauteng and Mpumalanga, South Africa, 2018–2022.

RTI Cases	Years, n (%), N = 1540						
	2018	2019	2020	2021	2022	Total	*p*-Value ^a^
**Viral Pathogens**							
HAdV	17 (1.10)	79 (5.12)	12 (0.78)	18 (1.17)	1 (0.06)	127 (8.24)	0.000 *
HboV	16 (1.03)	76 (4.92)	3 (0.19)	19 (1.23)	1 (0.06)	115 (7.46)	0.000 *
hCoV 229e	37 (2.40)	148 (9.61)	2 (0.12)	0 (0)	0 (0)	187 (12.14)	0.000 *
hCoV HKU1	1 (0.06)	22 (1.42)	1 (0.06)	0 (0)	0 (0)	24 (1.55)	0.000 *
hCoV NL63	1 (0.06)	19 (1.22)	2 (0.12)	7 (0.45)	0 (0)	29 (1.88)	0.077
hCoV OC43	4 (0.25)	8 (0.52)	8 (0.51)	10 (0.64)	0 (0)	30 (1.94)	0.447
EV	6 (0.38)	9 (0.58)	15 (0.97)	8 (0.52)	0 (0)	38 (2.46)	0.045
IAV	3 (0.19)	45 (2.92)	1 (0.06)	15 (0.97)	1 (0.06)	65 (4.22)	0.000 *
IAV H1N1	2 (0.12)	8 (0.51)	0 (0)	13 (0.84)	1 (0.06)	24 (1.55)	0.002 *
IBV	35 (2.27)	6 (0.39)	0 (0)	7 (0.45)	0 (0)	48 (3.11)	0.000 *
ICV	4 (0.25)	5 (0.32)	3 (0.19)	1 (0.06)	0 (0)	13 (0.84)	0.159
HMPV A&B	9 (0.58)	30 (1.94)	10 (0.64)	20 (1.29)	0 (0)	69 (4.48)	0.779
HPIV 1	6 (0.38)	13 (0.84)	4 (0.25)	3 (0.19)	0 (0)	26 (1.68)	0.165
HPIV 2	2 (0.13)	8 (0.52)	0 (0)	1 (0.06)	0 (0)	11 (0.71)	0.117
HPIV 3	9 (0.58)	23 (1.49)	1 (0.06)	21 (1.36)	0 (0)	54 (3.51)	0.001 *
HPIV 4	9 (0.58)	19 (1.23)	11 (0.71)	7 (0.45)	0 (0)	46 (2.98)	0.208
HpeV	3 (0.19)	11 (0.71)	6 (0.38)	3 (0.19)	0 (0)	23 (1.49)	0.507
*HRV*	3 (0.19)	49 (3.18)	114 (7.40)	61 (3.96)	1 (0.06)	228 (14.80)	0.000 *
RSV	12 (0.77)	30 (1.94)	48 (3.11)	86 (5.58)	0 (0)	176 (11.42)	0.000 *
**Bacterial Pathogens**							
*Haemophilus influenzae*	59 (3.83)	199 (12.92)	72 (4.67)	71 (4.61)	5 (0.32)	406 (26.33)	0.000 *
*Haemophilus influenzae b*	4 (0.25)	3 (0.19)	2 (0.12)	4 (0.25)	0 (0)	13 (0.84)	0.214
*Bordetella* spp.	7 (0.45)	124 (8.05)	4 (0.24)	1 (0.06)	0 (0)	136 (8.83)	0.000 *
*Chlamydophila pneumoniae*	47 (3.05)	152 (9.87)	0 (0)	0 (0)	0 (0)	199 (12.92)	0.000 *
*Klesiella pneumoniae*	62 (0.38)	203 (12.18)	26 (1.68)	22 (1.42)	1 (0.06)	314 (20.39)	0.000 *
Legionella	2 (0.13)	4 (0.25)	2 (0.12)	0 (0)	0 (0)	8 (0.52)	0.220
*Moraxella catarrhalis*	18 (1.17)	48 (3.11)	84 (5.45)	91 (5.90)	1 (0.06)	242 (15.71)	0.000 *
*Mycoplasma pneumoniae*	4 (0.25)	53 (3.44)	1 (0.06)	0 (0)	0 (0)	58 (3.76)	0.000 *
*Pneumocystis jirovecii*	12 (0.77)	34 (2.20)	27 (1.75)	15 (0.97)	0 (0)	88 (5.71)	0.057
Salmonella	1 (0.06)	4 (0.25)	1 (0.06)	0 (0)	0 (0)	6 (0.38)	0.394
*Staphylococcus aureus*	36 (2.33)	167 (10.84)	98 (6.36)	108 (7.01)	1(0.06)	410 (26.62)	0.067
*Streptococcus pneumoniae*	43 (2.79)	177 (11.49)	103 (6.68)	116 (7.53)	0 (0)	439 (28.50)	0.072

^a^ Pearson’s Chi-square test, (*p* ≤ 0.05); * statistically significant (*p* ≤ 0.05).

**Table 3:** Respiratory pathogens detected in hospitalised SARI patients relative to controls without respiratory symptoms in Gauteng and Mpumalanga, South Africa, 2018–2022.

Respiratory Pathogens	Cases (%); n = 1540	Controls (%); n = 336	Univariate Logistic Regression			Multivariate Logistic Regression Controlling for Age and Site		
			OR^a^	95% CI	*p*-Value	OR	95% CI	*p*-Value
**HAdV**	**127 (8.3)**	**12 (3.6)**	**2.41**	**1.32–4.41**	**0.004**	1.8	0.97–3.33	0.06
**HboV**	**115 (7.5)**	**11 (3.3)**	**2.37**	**1.26–4.45**	**0.007**	1.95	1.03–3.68	0.041
**HRV**	**228 (14.8)**	**22 (6.6)**	**2.46**	**1.56–3.88**	**0**	**2.01**	**1.32–3.32**	0.002 *
**RSV**	**176 (11.4)**	**3 (0.9)**	**14.24**	**4.52–44.85**	**0**	**11.36**	**3.59–35.97**	<0.001 *
**hCoV 229e**	**187 (12.1)**	**2 (0.6)**	**22.94**	**5.67–92.89**	**0**	**18.91**	**4.66–79.75**	<0.001 *
hCoV HKU1	24 (1.6)	2 (0.6)	2.63	0.62–11.17	0.191	2.66	0.62–11.47	0.188
hCoV NL63	29 (1.9)	4 (1.2)	1.58	0.55–4.53	0.392	1.23	0.43–3.57	0.7
hCoV OC43	30 (2.0)	4 (1.2)	1.64	0.57–4.68	0.356	1.42	0.49–4.10	0.519
EV	38 (2.5)	11 (3.3)	1.03	0.70–1.52	0.865	0.95	0.66–1.35	0.766
IAV	65 (4.2)	0 (0.0)	omitted			omitted		
IAV H1N1	24 (1.6)	2 (0.6)	2.63	0.62–11.17	0.19	2.16	0.50–9.27	0.301
**IBV**	**48 (3.1)**	**1 (0.3)**	**10.71**	**1.47–77.89**	**0.019**	**12.02**	**1.64–88.01**	0.014 *
ICV	13 (0.8)	4 (1.2)	0.7	0.23–2.17	0.539	0.69	0.22–2.18	0.53
**HMPV A&B**	**69 (4.5)**	**4 (1.2)**	3.87	1.40–10.68	0.009	**2.85**	**1.02–7.91**	0.045 *
HPIV 1	26 (1.7)	1 (0.3)	5.72	0.77–42.29	0.088	5.62	0.75–42.42	0.092
HPIV 2	11 (0.7)	0 (0.0)	omitted			omitted		
HPIV 3	54 (3.5)	5 (1.5)	2.39	0.95–6.02	0.064	1.68	0.66–4.27	0.278
HPIV 4	46 (3.0)	7 (2.1)	1.44	0.64–3.21	0.376	1.3	0.57–2.95	0.53
HPeV	23 (1.5)	0 (0.0)	omitted			omitted		
***Bordetella***	**136 (8.8)**	**1 (0.3)**	32.26	4.49–231.48	0.001	**23.04**	**3.20–165.91**	0.002 *
***Chlamydophila pneumoniae***	**199 (12.9)**	**1 (0.3)**	49.42	6.90–353.83	0	**38.44**	**5.36–275.85**	<0.001 *
***Haemophilus influenzae***	**406 (26.4)**	**34 (10.1)**	3.16	2.18–4.59	0	**2.69**	**1.84–0.92**	**0**
*Haemophilus influenzae b*	13 (0.8)	2 (0.6)	1.41	0.32–6.29	0.65	1.48	0.32–6.8	0.612
***Klebsiella pneumoniae***	**314 (20.4)**	**17 (5.1)**	4.78	2.89–7.90	0	**4.25**	**2.56–7.06**	<0.001 *
*Legionella*	8 (0.5)	0 (0.0)	omitted			omitted		
*Moraxella catarrhalis*	242 (15.7)	48 (14.3)	1.11	0.79–1.55	0.539	0.84	0.59–1.19	0.333
*Mycoplasma pneumoniae*	58 (3.8)	1 (0.3)	13.03	1.80–94.42	0.011	9.19	0.26–66.88	0.028
***Pneumocystis jirovecii***	**88 (5.7)**	**4 (1.2)**	5	1.82–13.72	0.002	**5.12**	**1.85–14.14**	0.002 *
*Salmonella*	6 (0.4)	2 (0.6)	0.65	0.13–3.23	0.598	0.64	0.12–3.3	0.591
***Staphylococcus aureus***	**410 (26.6)**	**56 (16.7)**	1.8	1.32–2.45	0	**1.66**	**1.21–2.26**	0.002 *
***Streptococcus pneumoniae***	**439 (28.5)**	**54 (16.1)**	2.07	1.51–2.82	0	**1.67**	**1.21–2.30**	0.002 *

Abbreviations: OR, odds ratio; CI, confidence interval (95%); ^a^ OR = 1, represents the detection of respiratory pathogens in cases and controls adjusted for age and site. * Statistically significant (*p* ≤ 0.05); respiratory pathogens in bold.

## Data Availability

No new data were created.
